# American Joker

**Published:** 1882-03

**Authors:** 


### THE AMERICAN JOKER.

*The Laugh Cure.*

A Merry Heart Doeth Good Like a Medicine.—*Solomon.*

From the February number of the *American Joker*—" a medical monthly!"—we quote: " It will astonish him [an inquirer] to learn that those who use the high potencies exclusively can be numbered upon the fingers of one hand. While those who make habitual use of the 30th do not number half a hundred. These, too, are men who run high-potency pharmacies, and write sensational books purporting to be medical *works*. Some of them are alleged teachers in homœopathic colleges. Few or more of them are active practitioners in actual competitive work against practitioners of other schools; and especially against those who rely upon the low potencies."

This is good, but better is to come: " The trash that has been dumped into homœopathic materia medica by Internationals, and their prototypes, the Korsakoffs, Bœnninghausens, Jahrs, Lutzes, *et. al.*, was spilt there against the will and without the consent of the discoverer of Homœopathy. The right-sided, the left-sided and slab-sided symptoms, along with the quieting of the child while being carried abou tunder cham. [is "cham." a new name for *parasol*, that child should be quiet while carried about *under* it?] and the content and cheerfulness of the Belladonna prover after stool, are samples of such rubbish. No homœopathic *practitioner* relies upon such so-called symptoms. * * * Finally, as to the declaration of Dr. Geary, that 'Hull's Jahr, Hering's inflated gas-bags, etc., have made a race of symptom doctors ignorant of every branch of medical and general knowledge,' I must again pronounce a most emphatic denial. The Herings, Lippes, Wilsons, Allens, taught and are still teaching false doctrines in the name of Homœopathy. These false doctrines cost the young practitioner some months of fruitless trial, discouragement and loss of practice and confidence." This writer asserts that ninety of every hundred homœopathic physicians agree with him. Then Homœopathy has indeed " gone to the dogs."

